# Associations between in-hospital bed occupancy and unplanned 72-h revisits to the emergency department: a register study

**DOI:** 10.1186/s12245-014-0025-4

**Published:** 2014-06-28

**Authors:** Mathias C Blom, Fredrik Jonsson, Mona Landin-Olsson, Kjell Ivarsson

**Affiliations:** 1 Department of Clinical Science Lund, Lund University, Hs 32, EA-blocket, Plan 2, Lund 22185, Sweden; 2 Department of Emergency, Helsingborg Hospital, S Vallgatan 5, Helsingborg 25187, Sweden

**Keywords:** Emergency medicine, Emergency medicine/organization and administration, Emergency medicine/statistics and numerical data, Bed occupancy

## Abstract

**Background:**

A possible downstream effect of high in-hospital bed occupancy is that patients in the emergency department (ED) who would benefit from in-hospital care are denied admission. The present study aimed at evaluating this hypothesis through investigating associations between in-hospital bed occupancy at the time of presentation in the ED and the probability for unplanned 72-hour (72-h) revisits to the ED among patients discharged at index. A second outcome was unplanned 72-h revisits resulting in admission.

**Methods:**

All visits to the ED of a 420-bed emergency hospital in southern Sweden between 1 January 2011 and 31 December 2012, which did not result in admission, death, or transfer to another hospital were included. Revisiting fractions were computed for in-hospital occupancy intervals <85%, 85% to 90%, 90% to 95%, 95% to 100%, 100% to 105%, and ≥105%. Multivariate models were constructed in an attempt to take confounding factors from, e.g., presenting complaints, age, referral status, and triage priority into account.

**Results:**

Included in the study are 81,878 visits. The fraction of unplanned 72-h revisits/unplanned 72-h revisits resulting in admission was 5.8%/1.4% overall, 6.2%/1.4% for occupancy <85%, 6.4%/1.5% for occupancy 85% to 90%, 5.8%/1.4% for occupancy 90% to 95%, 6.0%/1.6% for occupancy 95% to 100%, 5.4%/1.6% for occupancy 100% to 105%, and 4.9%/1.4% for occupancy ≥105%.

In the multivariate models, a trend to lower probability of unplanned 72-h revisits was observed at occupancy ≥105% compared to occupancy <95% (OR 0.88, CI 0.76 to 1.01). No significant associations between in-hospital occupancy at index and the probability of making unplanned 72-h revisits resulting in admission were observed.

**Conclusions:**

The lack of associations between in-hospital occupancy and unplanned 72-h revisits does not support the hypothesis that ED patients are inappropriately discharged when in-hospital beds are scarce. The results are reassuring as they indicate that physicians are able to make good decisions, also while resources are constrained.

## Background

High in-hospital bed occupancy has been associated with prolonged wait in the emergency department (ED) [[1],[2]], spread of hospital-associated infections [[3],[4]], and declining mental health among personnel [[5]]. Simulation studies suggest that periods of demand exceeding bed capacity are more frequent in systems with high-average occupancy [[6],[7]]. Pooling of resources appears to enable larger systems to operate at higher average levels of bed occupancy [[8]]. Application of the principles of queuing theory to hospital systems has shown that variability in admission rate or in length of stay (LOS) in hospital wards is associated with the presence and length of queues for in-hospital beds [[8],[9]].

Capacity planning in many hospital systems relies on average occupancy and average LOS, which makes them susceptible to overflow when these variables vary [[8]]. Several studies highlight the advantages of minimizing variability in elective volumes, to minimize overflows and increase efficiency [[10]–[13]].

Additional simulation studies have shown that performing discharges earlier in the day prevents collision of peak occupancy and peak demand for admissions, which results in lower daily peak and average bed occupancy [[14]–[16]].

A recent study undertaken by the authors revealed an association between high in-hospital bed occupancy and decreased probability of admission from the ED [[17]]. A possible downstream effect is that patients who benefit from in-hospital care are denied admission and instead receive care in the outpatient setting. The objective of the present study was to evaluate this hypothesis through investigating associations between in-hospital bed occupancy at the time of presentation in the ED and the probability of unplanned 72-h revisits to the ED, among patients discharged at their index visit.

## Methods

### Study design

The study was conducted as a retrospective register study, including all visits to the ED of a 420-bed emergency hospital in southern Sweden between 1 January 2011 and 31 December 2012, not resulting in admission, death, or transfer to another hospital. In order to avoid selection bias, no further selection was made.

### Setting

The ED of Helsingborg Hospital serves a population of around 250,000. Due to tourism, the population expands to nearly 300,000 during summer. It is one of the four emergency hospitals in the region of Skåne in southern Sweden. The annual ED census is around 60,000, with approximately 15% of patients arriving by ambulance. Patients are registered in the information system Patientliggaren® by a secretary upon arrival. Patients who arrive by ambulance or are referred by a physician gain access to the ED directly after registration. Other patients gain access to the ED in accordance with predefined guidelines, or are further evaluated by a nurse in primary triage. Patients could be referred elsewhere from primary triage (e.g., to primary care). Patients who gain access to the ED undergo secondary triage, which is performed by a nurse. The following is controlled upon secondary triage: Airway, respiratory rate, and SpO_2_ (pulse oximetry), heart rate, and blood pressure (non-invasive), alertness (Reaction Level Scale (RLS)), and body temperature. The four-level triage system ‘Medical emergency triage and treatment system’ (METTS) was used for secondary triage during the study period. The triage priority is registered in Patientliggaren® directly after the secondary triage. Only physicians may down-prioritize patients (Table 1).

**Table 1:** Overview of the four category triage system METTS

**Clinical parameter**	**Triage category**			
	**1 (red)**	**2 (orange)**	**3 (yellow)**	**4 (green)**
	Airway obstruction			
	Stridor			
Oxygen saturation	SpO_2_ < 90% with oxygen supply	SpO_2_ < 90% without oxygen supply	SpO_2_ 90% to 95% without oxygen supply	SpO_2_ > 95% without oxygen supply
Respiratory rate (breaths/min)	>30 or <8	>25		8 to 25
Pulse (beats/min)	Regular >130 or irregular >150	>120 or <40	>110 or <50	50 to 110
Systolic bp (mmHg)	<90			
Consciousness	Unconscious	RLS 2 to 3/somnolence	Disoriented	Alert
	Seizures	Temperature >41°C or <35°C	Temperature >38.5°C	Temperature 35°C to 38.5°C

METTS was used during the study period. The most urgent category from which the patient scores is selected.

Patients are directed to separate units for Surgery, Orthopedics, Medicine, and Otolaryngology in a triage-to-specialty model after the secondary triage. A complementary unit staffed by emergency physicians capable of handling various complaints except for psychiatric, otolaryngologic, ophthalmologic, and pediatric (medicine) complaints was introduced in 2010 and operates from 8 am to 11 pm daily. There are separate EDs for children with medical conditions (<18 years of age) and for patients with obstetric/gynecologic, psychiatric, or ophthalmologic complaints. Visits to these EDs were not included in the study. Patients with suspected hip fractures or ST elevation myocardial infarction diagnosed in the ambulance bypass the ED in fast tracks and were not included either. Hand surgery, neurosurgery, and thoracic surgery are not available in the hospital. The availability of endovascular surgery and percutaneous coronary intervention is limited from 5 pm to 08 am. Patients with such needs are referred to Skåne University Hospital and were not included in the study.

Physical ED records for patients who are advised to revisit the ED are stored at each specialty desk. Nurses indicate whether a visit is a planned revisit in Patientliggaren® upon patient arrival. Swedish national reimbursement systems are tied to a goal of 80% of visits with ED LOS ≤4 h. At in-hospital bed occupancy close to 100%, the hospital utilizes full-capacity protocols.

### Data sources

Data was retrieved from the ED information system Patientliggaren® and the in-hospital information system PASiS®. Data matching was performed by the hospital Informatics Unit using QlikView® software.

### Statistics

Occupancy was defined as the overall proportion of occupied beds in the hospital at whole-hour intervals. All patients registered in Patientliggaren® during an interval were assigned the same occupancy.

The proportion of visits resulting in an unplanned 72-h revisit was computed for in-hospital occupancy levels of <85%, 85% to 90%, 90% to 95%, 95% to 100%, 100% to 105%, and ≥105%. Subgroup analysis was performed for each specialty unit. Computations were repeated for unplanned 72-h revisits resulting in admission.

Adjusted analysis was performed in an attempt to take confounding factors into account, using binary logistic regression models. Perceived clinical significance governed the decision of screened predictors but was inevitably tainted by data availability. Screened variables were the following: specialty unit, presenting complaint at index, referral status at index, triage priority at index, age group, sex, index presentation on an intense shift, index presentation on a night shift and during weekends, leaving without being seen (LWBS) at index, entering ED via primary triage at index, time to physician at index, and in-hospital occupancy at index. The variable indicating presentation on an intense shift was constructed as a dichotomous variable indicating presentation on one of the 25% of shifts subject to most visits (adjusted for shift type and unit). Night shift was set from 12:00 mn to 08:00 am. Presenting complaint was constructed as a nominal variable indicating the ten most common complaints, using the remainder as reference.

The medicine unit was used as reference among the specialty units. Age was grouped into intervals 0 to 18 years, 18 to 40 years, 40 to 65 years, and ≥65 years. Age ≥65 years was used for reference. Youths in Sweden become of age at 18 and pension age is 65 years.

For the multivariate models, in-hospital occupancy was categorized as <85%, 85% to 90%, 90% to 95%, 95% to 100%, 100% to 105%, and ≥105%. The reference interval was set to <85%. Sensitivity analysis was performed using occupancy <95% as reference.

Predictors were tested for crude association with the outcome before entering the preliminary primary effects model. Associations weaker than *P* = 0.25, but of clinical importance were still included [[18]]. Multicollinearity testing was performed using Spearman correlation [[19]]. Selection of interaction terms screened for inclusion in the final models was governed by perceived clinical significance and made *a priori* to analysis. Variables were manually added to the models, rather than stepwise [[18]]. Missing data was indicated by a separate category and included in the models [[20]].

Model fit was evaluated through Nagelkerke’s *R*^2^. The association between each predictor and the outcome was addressed by the −2LL and the Wald statistics. The final models were the models with the highest explanatory value and the fewest number of predictors [[19]]. Additionally, the models were screened for influential cases by addressing standardized residuals and Cook’s distance.

Statistical analyses were performed in IBM® SPSS® Statistics 19. Data was anonymized before analysis. The Regional Ethical Review Board in Lund granted ethical approval for the study.

## Results

A total of 83,586 ED visits resulting in discharge were registered in Patientliggaren®. Of these, 81,878 did not result in referral to another hospital or death and were hence included in the study.

### Unadjusted analysis

**Figure 1:**
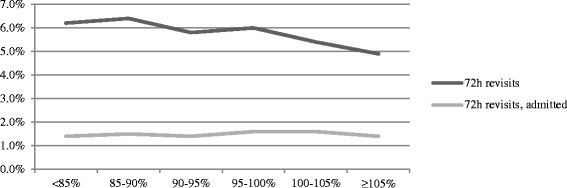
Overall proportion of unplanned 72-h revisits at different levels of in-hospital bed occupancy.

Out of the 81,878 cases, 4,753 cases resulted in an unplanned 72-h revisit, and 1,213 cases resulted in an unplanned 72-h revisit and admission. Proportions of unplanned 72-h revisits/unplanned 72-h revisits resulting in admission were 5.8%/1.5% overall, 6.2%/1.4% for occupancy <85%, 6.4%/1.5% for occupancy 85% to 90%, 5.8%/1.4% for occupancy 90% to 95%, 6.0%/1.6% for occupancy 95% to 100%, 5.4%/1.6% for occupancy 100% to 105%, and 4.9%/1.4% for occupancy ≥105% (Figure 1).

### Adjusted analysis

**Figure 2:**
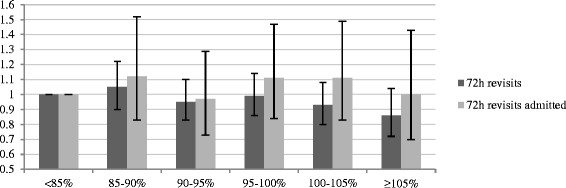
**Odds ratio for unplanned 72-h revisits.** Odds ratios (and confidence intervals) for making an unplanned 72-h revisit for patients discharged at different levels of in-hospital bed occupancy. Occupancy <85% was used for reference.

**Figure 3:**
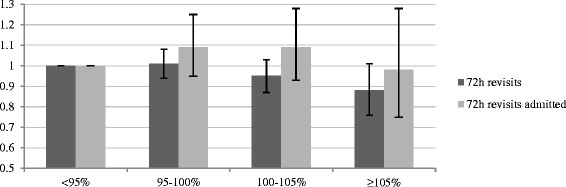
**Odds ratio for unplanned 72-h revisits.** Odds ratios (and confidence intervals) for making an unplanned 72-h revisit for patients discharged at different levels of in-hospital bed occupancy. Occupancy <95% was used for reference.

All predictors screened for inclusion in the multivariate models were included, except from time to physician, which was omitted as it violated the assumption of linearity in the logit [[19]]. No significant associations between in-hospital bed occupancy at the index visit and the probability for unplanned 72-h revisits was observed in the model using occupancy <85% as the reference. In the sensitivity analysis, a trend to lower odds for unplanned 72-h revisits was observed among patients being discharged from the ED at occupancy ≥105% relative to at <95%, OR 0.88 (CI 0.76 to 1.01, *P* = 0.062). No significant associations between in-hospital bed occupancy and the probability for unplanned 72-h revisits resulting in admission were seen in either model. A full account of the models (including coefficients of overall fit) is shown in Additional file 1 (Figures 2 and 3).

## Discussion

No significant association between making an index visit to the ED at times of high in-hospital bed occupancy and the probability for making an unplanned 72-h revisit was revealed in the multivariate model using occupancy <85% for reference. In the sensitivity analysis, a trend to lower odds for revisiting was observed among patients being discharged at occupancy ≥105% relative to at <95%. As the hospital rarely operates at occupancy <85%, the sensitivity analysis is considered most stable. The results are supported by the unadjusted analysis, which reveals that 4.9% of cases who were discharged at in-hospital occupancy ≥105% made an unplanned 72-h revisit, compared to 6.2% at occupancy <85%. The overall fraction of unplanned 72-h revisits of 5.8% is higher than the 1.4% to 5.5% described in other studies [[21]–[25]]. No associations between in-hospital bed occupancy and the probability of making an unplanned 72-h revisit resulting in admission were observed, either in the adjusted or unadjusted analyses. The 1.5% of discharged cases who made an unplanned revisit resulting in admission is somewhat higher than the 1.1% to 1.2% reported in other studies [[22],[23]].

One interpretation of the results is that the patients who are discharged from the ED at times of high in-hospital bed occupancy are not sicker than the patients being discharged at other times. Considering our previous results, which showed that the probability for being admitted from the ED is lower at times of high in-hospital occupancy [[17]], the present results suggest that ED physicians make good decisions, also when resources are constrained.

### Limitations

The Nagelkerke *R*^2^ coefficients (given in Additional file 1) indicate that the variables that were not available for study, e.g., diagnosis and co-morbidity, influenced the outcome in the adjusted analyses. This is also supported by the presence of some influential cases. The relatively large sample size is thought to have balanced some of this effect. As diagnosis and IPLOS vary across specialties, it might have been better to model occupancy in different in-hospital units separately. Unfortunately, this was not possible. The external validity of the results is limited, as the study was performed in a single hospital. The fact that some groups of patients are cared for in separate EDs (children with medical conditions and patients with obstetric/gynecologic, psychiatric, or ophthalmologic complaints) and others bypass the ED in fast tracks (patients with STEMI diagnosed in the ambulance and patients with suspected hip fractures) is important to note when comparing results to other EDs. Another limitation is that patients making an unplanned revisit to another ED in the region are not included in the study, but empirical knowledge suggests that this fraction is small. The authors also recognize that the chosen outcomes are not designed to evaluate the appropriateness of ED discharges. Their selection was motivated by frequent use in other studies and that the Swedish National Board for Health and Welfare made unplanned 72-h revisits subject to national follow-up from April 2013.

## Conclusions

The present study yields no support for the hypothesis that ED patients who are discharged from the ED at times of high in-hospital bed occupancy make more unplanned 72-h revisits to the ED than patients who are discharged when bed availability is better. The results are reassuring as they indicate that ED physicians make good decisions, also while resources are constrained. As the present study includes only two endpoints, the reader should interpret it carefully. The implementation of information systems capable of measuring more outcomes on the individual level and tracking patients on their journey across medical specialties is an essential step to allow more accurate description of potential risks.

## Competing interests

The authors declare that they have no competing interests.

## Authors’ contributions

MB, FJ, KI, and MLO designed the study protocol together. MB gathered and matched the data and carried out the statistical analyses and the writing of the draft. All authors proofread repeated versions of the manuscript. All authors read and approved the final manuscript.

## Additional file

## Supplementary Material

Additional file 1: **A full account of the models (including coefficients of overall fit). Table S2:** fraction of unplanned 72-h revisits (rev) for different levels of in-hospital occupancy. **Table S3:** data from adjusted analysis, with in-hospital bed occupancy <85% used for reference. **Table S4:** data from adjusted analysis, with in-hospital bed occupancy <95% used for reference. Click here for file
