# Supplementary material for: Associations between in-hospital bed occupancy and unplanned 72-h revisits to the emergency department: a register study
Source: Int J Emerg Med. 2014 Jun 28;7:25. doi: 10.1186/s12245-014-0025-4 (PMC4080705; doi:10.1186/s12245-014-0025-4)
Supplement: Additional file 1: — A full account of the models (including coefficients of overall fit). Table S2: fraction of unplanned 72-h revisits (rev) for different levels of in-hospital occupancy. Table S3: data from adjusted analysis, with in-hospital bed occupancy <85% used for reference. Table S4: data from adjusted analysis, with in-hospital bed occupancy <95% used for reference. [file s12245-014-0025-4-S1.docx]

Additional file

Table S2 – Fraction of unplanned 72-hour revisits (rev) for different levels of in-hospital occupancy.

|  | **In-hospital occupancy** | **<85%** | **85-90%** | **90-95%** | **95-100%** | **100-105%** | ≥**105%** | **Total** |
| --- | --- | --- | --- | --- | --- | --- | --- | --- |
| **All units**  N=81 878 | 72h rev (%) | 6.2 | 6.4 | 5.8 | 6.0 | 5.4 | 4.9 | 5.8 |
|  | 72h rev, admitted (%) | 1.4 | 1.5 | 1.4 | 1.6 | 1.6 | 1.4 | 1.5 |
| **Medicine unit**  N=18 340 | 72h rev (%) | 6.4 | 6.9 | 6.5 | 5.6 | 5.4 | 4.1 | 6.0 |
|  | 72h rev, admitted (%) | 1.9 | 1.9 | 1.8 | 1.7 | 2.0 | 1.5 | 1.8 |
| **Surgery unit**  N=18 594 | 72h rev (%) | 7.2 | 7.9 | 7.0 | 8.1 | 7.4 | 7.7 | 7.6 |
|  | 72h rev, admitted (%) | 1.8 | 2.1 | 1.9 | 2.5 | 2.7 | 2.6 | 2.3 |
| **Orthopaedics unit**  N=23 484 | 72h rev (%) | 5.2 | 5.1 | 4.5 | 4.6 | 3.8 | 3.3 | 4.4 |
|  | 72h rev, admitted (%) | 1.0 | 0.9 | 0.7 | 0.9 | 0.5 | 0.6 | 0.7 |
| **Emergency physician unit** N=11 574 | 72h rev (%) | 4.5 | 6.4 | 5.6 | 5.7 | 5.1 | 5.2 | 5.5 |
|  | 72h rev, admitted (%) | 1.4 | 2.0 | 1.5 | 1.7 | 1.5 | 1.7 | 1.6 |
| **Otolaryngology unit**  N=9 886 | 72h rev (%) | 8.0 | 5.4 | 5.2 | 5.9 | 6.1 | 5.2 | 5.8 |
|  | 72h rev, admitted (%) | 0.9 | 0.6 | 0.8 | 1.0 | 1.2 | 0.8 | 0.9 |

Table S3 – Data from adjusted analysis, in-hospital bed-occupancy <85% used for reference.

|  |  |  | **Unplanned 72h revisits**  Nagelkerke R^2^ = 0.031 | **Unplanned 72h revisits, admitted**  Nagelkerke R^2^ = 0.060 |
| --- | --- | --- | --- | --- |
| **Variable** |  | Frequency (%) | Odds ratio (95% conf. interval) | Odds ratio (95% conf. interval) |
| Main Complaint |  |  |  |  |
|  | Others | 38 366 (46.9) | reference | reference |
|  | Abdominal pain | 10 581 (12.9) | 1.45 (1.31 - 1.60) | 2.09 (1.75 - 2.50) |
|  | Chest pain | 4 673 (5.7) | 0.66 (0.56 - 0.77) | 0.75 (0.58 - 0.98) |
|  | Dyspnea | 2 498 (3.1) | 1.11 (0.94 - 1.32) | 0.92 (0.67 - 1.26) |
|  | Extremity pain | 5 354 (6.5) | 0.97 (0.84 - 1.12) | 0.48 (0.34 - 0.70) |
|  | Hand injury | 5 293 (6.5) | 0.71 (0.60 - 0.84) | 0.15 (0.08 - 0.30) |
|  | Extremity symtoms | 4 527 (5.5) | 1.14 (0.99 - 1.31) | 0.74 (0.55 - 1.01) |
|  | Head injury | 4 008 (4.9) | 0.55 (0.46 - 0.66) | 0.42 (0.28 - 0.63) |
|  | Foot injury | 3 278 (4.0) | 0.72 (0.59 - 0.88) | 0.40 (0.23 - 0.70) |
|  | Non specified disease | 1 645 (2.0) | 1.16 (0.95 - 1.41) | 1.29 (0.90 - 1.83) |
|  | Vertigo | 1 655 (2.0) | 1.14 (0.93 - 1.39) | 0.76 (0.48 - 1.18) |
| In-hospital bed occupancy |  |  |  |  |
|  | 0.00% - 84.99% | 4 048 (4.9) | reference | reference |
|  | 85.00% - 89.99% | 10 720 (13.1) | 1.05 (0.90 - 1.22) | 1.12 (0.83 - 1.52) |
|  | 90.00% - 94.99% | 21 711 (26.5) | 0.95 (0.83 - 1.10) | 0.97 (0.73 - 1.29) |
|  | 95.00% - 99.99% | 23 632 (28.9) | 0.99 (0.86 - 1.14) | 1.11 (0.84 - 1.47) |
|  | 100.00% - 104.99% | 16 733 (20.4) | 0.93 (0.80 - 1.08) | 1.11 (0.83 - 1.49) |
|  | ≥105.00% | 5 034 (6.1) | 0.86 (0.72 - 1.04) | 1.00 (0.70 - 1.43) |
| Discharge  specialty |  |  |  |  |
|  | Medicine | 18 340 (22.4) | reference | reference |
|  | Surgery | 18 594 (22.7) | 1.15 (1.03 - 1.28) | 0.99 (0.82 - 1.20) |
|  | Ortho. | 23 484 (28.7) | 0.88 (0.79 - 0.98) | 0.75 (0.61 - 0.92) |
|  | Otolaryng. | 9 886 (12.1) | 1.16 (1.03 - 1.31) | 0.69 (0.53 - 0.88) |
|  | Emergency medicine | 11 574 (14.1) | 0.99 (0.89 - 1.10) | 0.94 (0.78 - 1.15) |
| Age group (years) |  |  |  |  |
|  | 0 - 17.99 | 14 379 (17.6) | 0.74 (0.67 - 0.82) | 0.39 (0.32 - 0.48) |
|  | 18 - 39.99 | 25 242 (30.8) | 0.75 (0.69 - 0.82) | 0.40 (0.34 - 0.46) |
|  | 40 - 64.99 | 24 298 (29.7) | 0.86 (0.80 - 0.93) | 0.58 (0.50 - 0.66) |
|  | 65 - | 17 959 (21.9) | reference | reference |
| Referral status |  |  |  |  |
|  | Not  referred | 63 275 (77.3) | reference | reference |
|  | Referred | 13 175 (16.1) | 0.73 (0.66 - 0.80) | 0.76 (0.64 - 0.91) |
|  | Missing | 5 428 (6.6) | 1.36 (1.21 - 1.53) | 1.21 (0.95 - 1.55) |
| Triage priority 1 |  | 1 161 (1.4) | 0.95 (0.74 - 1.21) | 1.50 (1.01 - 2.23) |
| Triage priority 2 |  | 7 818 (9.5) | 1.20 (1.09 - 1.32) | 1.68 (1.43 - 1.97) |
| Triage priority 3 |  | 46 323 (56.6) | reference | reference |
| Triage priority 4 |  | 25 205 (30.8) | 0.83 (0.78 - 0.90) | 0.75 (0.65 - 0.87) |
| Triage priority missing |  | 1 371 (1.7) | 1.61 (1.35 - 1.92) | 1.45 (1.00 - 2.12) |
| Intense shift |  | 24 250 (29.6) | 1.07 (1.00 - 1.14) | 0.97 (0.85 - 1.10) |
| Night shift |  | 9 281 (11.3) | 1.15 (1.05 - 1.26) | 0.92 (0.77 - 1.10) |
| LWBS |  | 2 332 (2.8) | 2.28 (1.98 - 2.63) | 1.42 (1.02 - 1.97) |
| Weekend |  | 22 235 (27.2) | 1.09 (1.02 - 1.17) | 1.05 (0.92 - 1.21) |
| Sex | Female | 40 281 (49.2) | reference | reference |
|  | Male | 41 597 (50.8) | 1.18 (1.11 - 1.26) | 1.26 (1.12 - 1.41) |
| Entered via triage |  | 16 407 (20.0) | 0.86 (0.80 - 0.93) | 0.71 (0.59 - 0.84) |
| Constant |  |  | 0.066 | 0.026 |

Table S4 – Data from adjusted analysis, in-hospital bed-occupancy <95% used for reference.

|  |  |  | **Unplanned 72h revisits**  Nagelkerke R^2^ = 0.031 | **Unplanned 72h revisits, admitted**  Nagelkerke R^2^ = 0.060 |
| --- | --- | --- | --- | --- |
| **Variable** |  | Frequency (%) | Odds ratio (95% conf. interval) | Odds ratio (95% conf. interval) |
| Main Complaint |  |  |  |  |
|  | Others | 38 366 (46.9) | reference | reference |
|  | Abdominal pain | 10 581 (12.9) | 1.45 (1.31 - 1.60) | 2.09 (1.75 - 2.50) |
|  | Chest pain | 4 673 (5.7) | 0.66 (0.56 - 0.77) | 0.75 (0.58 - 0.98) |
|  | Dyspnea | 2 498 (3.1) | 1.11 (0.94 - 1.32) | 0.92 (0.67 - 1.26) |
|  | Extremity pain | 5 354 (6.5) | 0.97 (0.84 - 1.12) | 0.48 (0.34 - 0.70) |
|  | Hand injury | 5 293 (6.5) | 0.71 (0.60 - 0.84) | 0.15 (0.08 - 0.30) |
|  | Extremity symtoms | 4 527 (5.5) | 1.14 (0.99 - 1.31) | 0.74 (0.55 - 1.01) |
|  | Head injury | 4 008 (4.9) | 0.55 (0.46 - 0.66) | 0.42 (0.28 - 0.63) |
|  | Foot injury | 3 278 (4.0) | 0.72 (0.59 - 0.88) | 0.40 (0.23 - 0.70) |
|  | Non specified disease | 1 645 (2.0) | 1.16 (0.95 - 1.41) | 1.29 (0.90 - 1.84) |
|  | Vertigo | 1 655 (2.0) | 1.14 (0.93 - 1.39) | 0.76 (0.48 - 1.18) |
| In-hospital bed occupancy |  |  |  |  |
|  | 00.00% - 94.99% | 36 479 (44.6) | reference | reference |
|  | 95.00% - 99.99% | 23 632 (28.9) | 1.01 (0.94 - 1.08) | 1.09 (0.95 - 1.25) |
|  | 100.00% - 104.99% | 16 733 (20.4) | 0.95 (0.87 - 1.03) | 1.09 (0.93 - 1.28) |
|  | ≥105.00% | 5 034 (6.1) | 0.88 (0.76 - 1.01) | 0.98 (0.76 - 1.28) |
| Discharge  specialty |  |  |  |  |
|  | Medicine | 18 340 (22.4) | reference | reference |
|  | Surgery | 18 594 (22.7) | 1.15 (1.03 - 1.28) | 0.99 (0.82 - 1.21) |
|  | Ortho. | 23 484 (28.7) | 0.88 (0.79 - 0.98) | 0.75 (0.61 - 0.93) |
|  | Otolaryng. | 9 886 (12.1) | 1.16 (1.03 - 1.31) | 0.69 (0.53 - 0.88) |
|  | Emergency medicine | 11 574 (14.1) | 0.99 (0.89 - 1.10) | 0.94 (0.78 - 1.15) |
| Age group (years) |  |  |  |  |
|  | 0 - 17.99 | 14 379 (17.6) | 0.75 (0.67 - 0.83) | 0.39 (0.32 - 0.48) |
|  | 18 - 39.99 | 25 242 (30.8) | 0.75 (0.69 - 0.82) | 0.40 (0.34 - 0.47) |
|  | 40 - 64.99 | 24 298 (29.7) | 0.86 (0.80 - 0.93) | 0.58 (0.50 - 0.66) |
|  | 65 - | 17 959 (21.9) | reference | reference |
| Referral status |  |  |  |  |
|  | Not referred | 63 275 (77.3) | reference | reference |
|  | Referred | 13 175 (16.1) | 0.73 (0.66 - 0.80) | 0.76 (0.64 - 0.91) |
|  | Missing | 5 428 (6.6) | 1.36 (1.21 - 1.53) | 1.21 (0.95 - 1.55) |
| Triage priority 1 |  | 1 161 (1.4) | 0.95 (0.74 - 1.21) | 1.50 (1.01 - 2.23) |
| Triage priority 2 |  | 7 818 (9.5) | 1.20 (1.09 - 1.32) | 1.68 (1.43 - 1.98) |
| Triage priority 3 |  | 46 323 (56.6) | reference | reference |
| Triage priority 4 |  | 25 205 (30.8) | 0.84 (0.78 - 0.90) | 0.75 (0.65 - 0.88) |
| Missing |  | 1 371 (1.7) | 1.61 (1.35 - 1.92) | 1.45 (1.00 - 2.12) |
| Intense shift |  | 24 250 (29.6) | 1.07 (1.00 - 1.14) | 0.97 (0.85 - 1.10) |
| Night shift |  | 9 281 (11.3) | 1.15 (1.05 - 1.25) | 0.92 (0.77 - 1.10) |
| LWBS |  | 2 332 (2.8) | 2.28 (1.98 - 2.64) | 1.42 (1.02 - 1.97) |
| Weekend |  | 22 235 (27.2) | 1.10 (1.02 - 1.17) | 1.06 (0.92 - 1.21) |
| Sex | Female | 40 281 (49.2) | reference | reference |
|  | Male | 41 597 (50.8) | 1.18 (1.11 - 1.26) | 1.26 (1.12 - 1.41) |
| Entered via triage |  | 16 407 (20.0) | 0.86 (0.80 - 0.93) | 0.71 (0.59 - 0.84) |
| Constant |  |  | 0.065 | 0.027 |
